# Draft Genome Sequence of Novosphingobium sp. Strain MBES04, Isolated from Sunken Wood from Suruga Bay, Japan

**DOI:** 10.1128/genomeA.01373-14

**Published:** 2015-01-15

**Authors:** Yukari Ohta, Shinro Nishi, Kiwa Kobayashi, Taishi Tsubouchi, Kagami Iida, Akiko Tanizaki, Kanako Kurosawa, Akiko Adachi, Mizue Nishihara, Reona Sato, Ryoichi Hasegawa, Yuji Hatada

**Affiliations:** Japan Agency for Marine-Earth Science and Technology, Yokosuka-shi, Kanagawa, Japan

## Abstract

This report describes the draft genome sequence of Novosphingobium sp. strain MBES04, isolated from sunken wood from Suruga Bay, Japan, which is capable of degrading a wide range of lignin-related aromatic monomers. The draft genome sequence contains 5,361,448 bp, with a G+C content of 65.4%.

## GENOME ANNOUNCEMENT

The genus Novosphingobium is an important group involved in the biodegradation of a wide range of mono- and polycyclic aromatic compounds (1–3). These compounds are widely known as components of fossil fuel and share structural futures with lignin, which is produced in terrestrial plants and is the most abundant aromatic biomass on Earth (4). The evolution of microbial pathways for using a wide variety of aromatic molecules as carbon sources was likely triggered by naturally produced aromatic compounds during lignin decomposition by some microorganisms (5). We recently isolated a marine Novosphingobium strain, isolated from sunken wood recovered from a depth of 260 m in Suruga Bay, off the Pacific coast of Shizuoka Prefecture, central Japan (6). The strain was found to metabolize a wide range of lignin-related aromatic monomers. To gain deeper insight into the genetic mechanism and evolution in the utilization of terrestrial plant-derived compounds developed in marine bacteria, we sequenced the draft genomic sequence of Novosphingobium sp. strain MBES04.

The draft genome of strain MBES04 was sequenced using both 454 GS FLX (454 Life Sciences) and Ion Torrent PGM (Life Technologies). The 454 GS FLX sequencing revealed 142,389 mate-pair reads and 62,888,162 nucleotide bases, with an average read length of 442 bp, whereas the Ion Torrent PGM sequencing revealed 2,652,670 reads and 873,653,659 nucleotide bases, with an average read length of 329 bp. Assembly using Newbler version 2.6 (454 Life Sciences) generated 124 contigs contained in 33 scaffolds (maximum, 3,129,454 bp; minimum, 553 bp). The draft genome consists of 5,361,448 nucleotides with a G+C content of 65.4%. Based on the genomic data, 4,728 coding sequences (CDSs) were predicted by GeneMark (7) and annotated by a BLAST tools search against the GenBank nonredundant protein database (NR) and Kyoto Encyclopedia of Genes and Genomes (KEGG). In addition, 50 tRNAs and 3 rRNAs were identified with the tRNAscan-SE 1.23 (8) and RNAmmer 1.2 (9) servers, respectively.

The draft genome of strain MBES04 showed the presence of a benzoate-degrading gene cluster with the presence of benzoate transporter proteins, 2-chlorobenzoate 1,2-dioxygenase, and genes for the catechol branch of the β-ketoadipate pathway (10). Additionally, genes known to be involved in aromatic compound degradation, including *p*-hydroxybenzoate 3-monooxygenase and vanillate monooxygenase, were also found in the scattered position of the genome. Interestingly, this strain had three copies of the protocatechuate 4,5-dioxygenase alpha and beta subunits for the metacleavage pathway of protocatechuate, which is a key compound in lignin-related aromatic compound-degrading pathways (11). Furthermore, the multiple hemicellulose-degrading enzymes, such as xylan 1,4-β-xylosidase, α-*N*-arabinofuranosidase, and the other glucoside hydrolases were classified into glycoside hydrolase families 2, 16, and 43 (see http://www.cazy.org/Glycoside-Hydrolases.html).

### Nucleotide sequence accession numbers.

This whole-genome shotgun project has been deposited at DDBJ/EMBL/GenBank under the accession no. BBNP00000000. The 124 contigs have been deposited under the accession numbers BBNP01000001 to BBNP01000124.

## References

[1] Aylward FO, McDonald BR, Adams SM, Valenzuela A, Schmidt RA, Goodwin LA, Woyke T, Currie CR, Suen G, Poulsen M 2013 Comparison of 26 sphingomonad genomes reveals diverse environmental adaptations and biodegradative capabilities. Appl Environ Microbiol 79:3724–3733. doi:10.1128/AEM.00518-13. 23563954 PMC3675938

[2] Looper JK, Cotto A, Kim BY, Lee MK, Liles MR, Ní Chadhain SM, Son A 2013 Microbial community analysis of deepwater horizon oil-spill impacted sites along the Gulf Coast using functional and phylogenetic markers. Environ Sci Process Impacts 15:2068–2079. doi:10.1039/c3em00200d. 24061682

[3] Sohn JH, Kwon KK, Kang JH, Jung HB, Kim SJ 2004 Novosphingobium pentaromativorans sp. nov., a high-molecular-mass polycyclic aromatic hydrocarbon-degrading bacterium isolated from estuarine sediment. Int J Syst Evol Microbiol 54:1483–1487. doi:10.1099/ijs.0.02945-0. 15388699

[4] Sarakanen KV, Ludwig CH 1971 Lignins, occurrence, formation, structure, and reactions. Wiley Interscience, New York, NY.

[5] Linger JG, Vardon DR, Guarnieri MT, Karp EM, Hunsinger GB, Franden MA, Johnson CW, Chupka G, Strathmann TJ, Pienkos PT, Beckham GT 2014 Lignin valorization through integrated biological funneling and chemical catalysis. Proc Natl Acad Sci U S A 111:12013–12018. doi:10.1073/pnas.1410657111. 25092344 PMC4143016

[6] Ohta Y, Nishi S, Haga T, Tsubouchi T, Hasegawa R, Konishi M, Nagano Y, Tsuruwaka Y, Shimane Y, Mori K, Usui K, Suda E, Tsutsui K, Nishimoto A, Fujiwara Y, Maruyama T, Hatada Y 2012 Screening and phylogenetic analysis of deep-sea bacteria capable of metabolizing lignin-derived aromatic compounds. Open J Mar Sci 2:177–187. doi:10.4236/ojms.2012.24021.

[7] Besemer J, Borodovsky M 2005 GeneMark: web software for gene finding in prokaryotes, eukaryotes and viruses. Nucleic Acids Res 33:W451–W454. doi:10.1093/nar/gki487. 15980510 PMC1160247

[8] Lowe TM, Eddy SR 1997 tRNAscan-SE: a program for improved detection of transfer RNA genes in genomic sequence. Nucleic Acids Res 25:955–964. doi:10.1093/nar/25.5.0955. 9023104 PMC146525

[9] Lagesen K, Hallin P, Rødland EA, Staerfeldt HH, Rognes T, Ussery DW 2007 RNAmmer: consistent and rapid annotation of ribosomal RNA genes. Nucleic Acids Res 35:3100–3108. doi:10.1093/nar/gkm160. 17452365 PMC1888812

[10] Fuchs G, Boll M, Heider J 2011 Microbial degradation of aromatic compounds—from one strategy to four. Nat Rev Microbiol 9:803–816. doi:10.1038/nrmicro2652. 21963803

[11] Masai E, Katayama Y, Fukuda M 2007 Genetic and biochemical investigations on bacterial catabolic pathways for lignin-derived aromatic compounds. Biosci Biotechnol Biochem 71:1–15. doi:10.1271/bbb.60437. 17213657

